# Oral disorders related to acromegaly

**DOI:** 10.11604/pamj.2019.34.96.19873

**Published:** 2019-10-16

**Authors:** Akram Belmehdi, Saliha Chbicheb

**Affiliations:** 1 Oral Surgeon, Dental Center of Treatment and Diagnosis, Ibn Sina Hospital, Rabat, Morocco; 2 Oral surgery department, Faculty of Dentistry of Rabat, Mohammed V University, Morocco

**Keywords:** Oral disorders-acromegaly, growth hormones

## Abstract

Acromegaly is a rare disease caused due to hyper secretion of growth hormone. Most of the cases of acromegaly are caused by pituitary adenoma which can be microadenoma or macroadenomas. This condition has a tendency toward overgrowth of the mandible, maxillary widening, tooth separation and skeletal malocclusion which compromises the aesthetics of an individual. Hence dentists have a role in diagnosing this disorder. The aim of this paper is to present a case report of acromegaly.

## Introduction

The term ''acromegaly'' (acrogigantism) has its origin from the Greek words ''akros'' which means extremities and ''mega'' which means big. The disorder has been cited in literature since ancient ages, but the pathology of pituitary gland that leads to this condition was the first described by an Italian anatomist, Andrea Verga, in 1864 [1]. Acromegaly is a rare and underdiagnosed disease that results from the overproduction of growth hormone (GH) and insulin-like growth factor 1 (IGF-1) [2,3]. The disorder is known as acromegaly if it occurs during adulthood (around the age of 40), whereas gigantism is the childhood counterpart of the same disorder [4]. More than 95% of patients with acromegaly harbor a GH-secreting pituitary adenoma (somatotropinoma) arising from somatotroph cells, leading to GH and IGF-1 hypersecretion [5]. In less than 5% of cases, excess growth hormone releasing hormone (GHRH) secretion from a hypothalamic tumor or a neuroendocrine tumor (usually from lung or pancreas origin) may lead to somatotroph hyperplasia and acromegaly [3,6]. Acromegaly being a rare disease, it incidence is estimated to be 3 to 4 cases per million per year [7], although there have been only 100 reported cases of gigantism in medical literature [4,8]. The resultant clinical presentations are characterized by the coarse facial features, large, spade shaped hands, and enlarged feet resulting from soft tissue swelling and bony enlargement. These clinical manifestations may range from minimal physical changes to severe disfiguring features. The early changes may go unnoticed by patients due to the inconspicuous nature of the disease [9]. Earlier diagnosis of acromegaly could be enabled by greater awareness of acromegaly symptoms and greater collaboration between general practitioners (GPs) and dentists. There is no formal integration of care between dentists and GPs: patients usually attend their GP for health-related symptoms and are sometimes referred to a hospital consultant if needed. Hence, adequate knowledge about the signs and symptoms form the key to arrive at the diagnosis [10].

## Patient and observation

**Figure 1:**
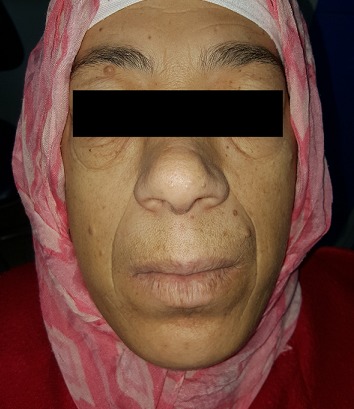
Facial view: enlarged lips and nose

**Figure 2:**
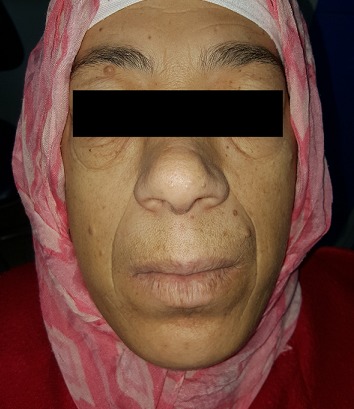
Fingers appear thickened and stubby

**Figure 3:**
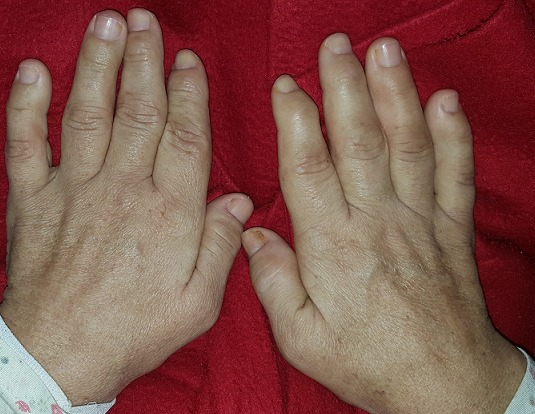
Macroglossia

**Figure 4:**
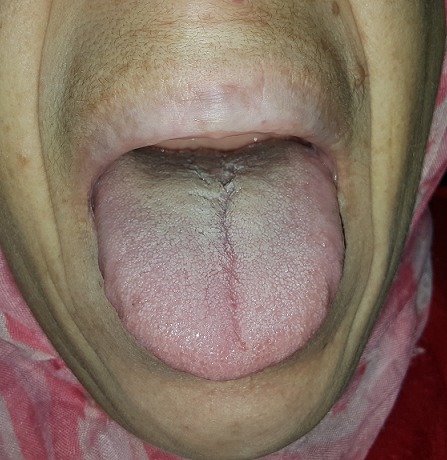
Intraoral view

**Figure 5:**
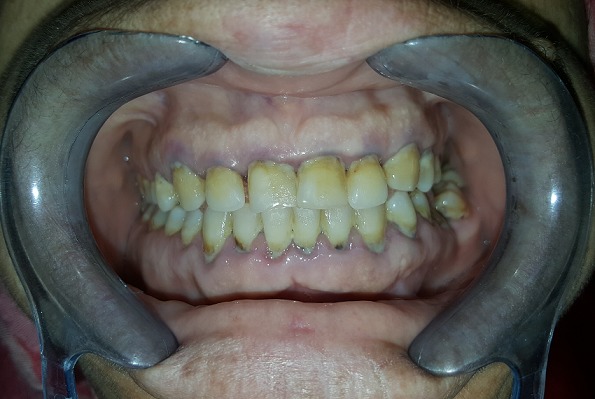
Orthopantomogram showing a generalized moderate bone loss, root fragment of 36 and hypercemoentosis (16,27,37 and 46)

A 43-year-old female patient reported to the Department of Oral Surgery with a chief complaint of painful episodes in the left mandibular area, halitosis and increase in the size of lips and tongue since 8 years. Evaluation of his medical history revealed she had been diagnosed with acromegaly at the age of 40. She gives a history of gradual decrease in eyesight and frequent headaches episodes since past 2 years. She was also diagnosed with hypertension (140/90 mmHg), hyperglycemia and hyperthyroidism. Extra oral clinical examination revealed classic features of acromegaly including facial asymmetry, thickened lips, mild mandibular prognathism, and retrusion of maxilla, proptosis, prominent nose and supraorbital ridges (Figure 1). Skin over the forehead appeared thickened. Fingers and toes appeared large and broad (Figure 2). Intraoral examination revealed, macroglossia (Figure 3), Angle's class-III malocclusion, the first left molar 36 was decayed and poor oral hygiene (Figure 4). The patient was subjected to orthopantomogram radiographic examination which revealed generalized moderate bone loss, root fragment of 36 and hypercemoentosis (16,27,37 and 46) (Figure 5). Considering the patient's uncontrolled diabetes and hypertension, dental treatment was deferred and he was referred to an endocrinologist for further evaluation.

## Discussion

Acromegaly is an acquired progressive disorder, characterized by disfigurement, mainly involving the face and extremities, but also multi-organ involvement, that leads to systemic manifestations. The disease is manifested due to the excessive production of GH post epiphyseal closure. This excessive GH release is attributable to benign pituitary tumor (adenoma) in more than 90% of cases [11]. GH hypersecretion may primary be caused by pituitary adenomas and secondary by pituitary hypersecretion owing to excessive production of hypothalamic GH releasing hormones or genetic disorders, such as multiple endocrine neoplasia type-1, McCune-Albright syndrome or carney complex [4]. Acromegaly affects both males and females equally and the average age at diagnosis ranges from 40 to 50 years. Patients under the age 20 account for up to 5% of cases [7,12]. The clinical features of acromegaly are due to local effects of an expanding pituitary mass, as well as to the direct and indirect effects of excessive secretion of GH and IGF-1, which can lead to systemic complications and impaired quality of life (QoL) [2,3]. These clinical manifestations range from subtle signs of acral overgrowth, soft-tissue swelling, arthralgias, jaw prognathism, mild hyperglycemia, menstrual disturbances, erectile dysfunction and hyperhidrosis to facial and skeletal disfigurement, florid osteoarthritis, severe headache, sleep apnea, severe hypertension, diabetic ketoacidosis, and respiratory and cardiac failure [2,3]. The appearance of a somatotropinoma in young patients before the closure of epiphyseal bone results in accelerated growth and gigantism [3,13,14]. Several changes in different vital organs are induced by this rare systemic. Among them is a change in occlusion that can bring the patient to the dentist first. Dental professionals may be the first healthcare providers to see the signs [15]. Craniofacial changes are characteristic of this disease and may involve facial skin, extraoral and intraoral soft and hard tissues [16]. Glycosaminoglycan deposition and increased collagen production by connective tissue lead to skin thickening [17]. Facial features of Acromegaly patients are characteristic, and generally look alike in this respect: The nose is broadened and thickened, the malar bone becomes prominent, the lips are thick, and the facial lines are marked. Mandibular prognathism and jaw thickening are due to deposition of periosteal bone in response to the excess growth hormone [2,3,16,18]. Other intraoral manifestations are teeth separation, aperthognathia, malocclusion, macroglossia, hypertrophy of palatal tissues which may cause or accentuate sleep apnea, buccal tipping of the teeth due to enlarged tongue [3,4,9,18]. The deep voice in acromgaly patients is due to hypertrophy of the sinuses, along with laryngeal hypertrophy. Dental radiology investigation may demonstrate large pulp chambers (taurodontism) and hypercementosis on the roots of posterior teeth, as seen in our patient [3,4,9,18]. According to the morphologic analysis study conducted in Japan by Takakura *et al*. [19], male patients tended to demonstrate downward mandibular advancement and crossbite, while females showed extension of the ascending ramus, downward displacement of mandible, bimaxillary alveolar protrusion, and edge-to-edge bite. The disease also has rheumatologic, cardiovascular, respiratory, and metabolic consequences which determine its prognosis [9].

In a retrospective survey study conducted by Kreitschmann-Andermahr *et al*. [20] he shows that four of five patients were affected by any oro-dental pathologies at any time during the course of the disease, most frequently consisting of macroglossia, teeth spacing, mandibular growth, and prognathism. All symptoms were reduced significantly after the treatment of acromegaly. About half of the patients required dental assistance, but less than 5% of the patients were referred to a specialist due to suspected acromegaly. Oral manifestations of hyperpituitarism may give clues to serious systemic problems, complications of which may include cardio-vascular complications which are the most common cause of morbidity and early mortality. Hypertension is caused by increased sodium reabsorption in the kidney and endothelial dysfunction, and asymptomatic cardiac hypertrophy occurs in 60% of untreated acromegalic patients [3,10]. Metabolic complications include impaired glucose intolerance due to growth hormone-induced insulin resistance and lipid abnormalities like hypertriglyceridemia. Muskuloskeletal complications and arthropathy along with other endocrinal disturbances including benign thyroid overgrowth, hypogonadism, and gonadal dysfunction may also be observed as associated complications [9,10]. Early intervention by endocrinologists to correct the underlying hormonal disorder is required to prevent further growth deterioration or imbalance. Treatment plan include surgery, radiotherapy, and medical therapy. In some cases, surgical intervention is needed to eliminate the causative factor (such as pituitary tumors) or correct disfigurement [4]. Radiotherapy is effective in controlling tumor growth and GH secretion; however, achievement of biochemical targets may take a long time almost up to a decade, and some safety issues have been raised with this treatment modality [2]. More recently, medical management has been increasingly used as primary treatment in selected patients unsuitable for surgery. Dopaminergic agonists like cabergoline, somatostatin analogues like octreotide, and GH receptor antagonists like pegvisomant, are the clinically available medical therapies for management of acromegaly [2]. Surgical therapy includes transsphenoidal approach and transnasal endoscopic approach frontotemporal craniotomy [2,6]. Orthodontic consultations for correction of hypognathia or hypergnathia and malocclusion should be pursued as early as possible. Prosthodontic work and dental implants can help those with teeth agenesis and amelogenesis imperfecta. Those with signs and symptoms of sleep apnea should be referred for pulmonology or ear, nose, and throat examination and, if required, sleep test (polysomnography) can be performed. Dentist can help those with mild to moderate sleep apnea by constructing dental sleep appliances. Dental management may be complicated by blindness, diabetes mellitus, hypertension, cardiomyopathicdys arrhythmias, or hypopituitarism [4].

## Conclusion

Acromegaly is a rare disease caused due to excessive secretion of growth hormone and insulin like growth factor type 1 mostly due to pituitary adenoma. Clinical features like prognathism, frontal bossing, macroglossia, spade like hands, thick heel pad, thickening of lips, sweating and headache as seen in this patient. Given the rarity of this pattern, the aim of presenting this clinical case is to enrich the medical literature with a new clinical case of acromegaly. The purpose is to allow an early detection and diagnosis by dentist which sometimes prevent a potentially life-threatening event, and it is very important for both endocrinologists and dentists to communicate with each other to order diagnostic tests, evaluate the results and create, administer and monitor treatment plans, and medications designed to address the problems.

## Competing interests

The authors declare no competing interests.

## References

[1] Brennan, Jackson IT, Keller EE, Laws ER, Sather AH (1985). Multidisciplinary management of acromegaly and its deformities. JAMA.

[2] Melmed S (2006). Acromegaly. N Engl J Med.

[3] Vilar L, Vilar CF, Lyra R, Lyra R, Naves LA (2017). Acromegaly: clinical features at diagnosis. Pituitary.

[4] Farag AM (2017). Head and neck manifestations of endocrine disorders. Atlas Oral Maxillofac Surg Clin North Am.

[5] Katznelson L, Laws ER, Melmed S, Molitch ME, Murad MH, Utz A (2014). Endocrine Society Acromegaly: an endocrine society clinical practice guideline. J Clin Endocrinol Metab.

[6] Borson-Chazot F, Garby L, Raverot G, Claustrat F, Raverot V, Sassolas G (2012). Acromegaly induced by ectopic secretion of GHRH: a review 30 years after GHRH discovery. Ann Endocrinol.

[7] Etxabe J, Gaztambide S, Latorre P, Vazquez JA (1993). Acromegaly: an epidemiological study. J Endocrinol Invest.

[8] Sotos JF (1996). Overgrowth; hormonal causes. Clin Pediatr (Phila).

[9] Atreja G, Atreja SH, Jain N, Sukhija U (2012). Oral manifestations in growth hormone disorders. Indian J Endocr Metab.

[10] Zarool-Hassan R, Conaglen HM, Conaglen JV, Elston MS (2016). Symptoms and signs of acromegaly: An ongoing need to raise awareness among healthcare practitioners. J Prim Health Care.

[11] Chanson P, Salenave S (2008). Acromegaly. Orphanet J Rare Dis.

[12] Holdaway IM, Rajasoorya C (1999). Epidemiology of acromegaly. Pituitary.

[13] Capatina C, Wass JA (2015). 60 years of neuroendocrinology: acromegaly. J Endocrinol.

[14] Daughaday WH (1992). Pituitary gigantism. Endocrinol Metab Clin North Am.

[15] Trivellin G, Daly AF, Faucz FR, Yuan B, Rostomyan L, Larco DO (2014). Gigantism and acromegaly due to Xq26 microduplications and GPR101 mutation. N Engl J Med.

[16] Daly AF, Yuan B, Fina F, Caberg JH, Trivellin G, Rostomyan L (2016). Somatic mosaicism underlies X-linked acrogigantism (XLAG) syndrome in sporadic male subjects. Endocr Relat Cancer.

[17] Naves LA, Daly AF, Dias LA, Yuan B, Zakir JC, Barra GB (2016). Aggressive tumor growth and clinical evolution in a patient with X-linked acro-gigantism syndrome. Endocrine.

[18] Salenave S, Boyce AM, Collins MT, Chanson P (2014). Acromegaly and McCune-Albright syndrome. J Clin Endocrinol Metab.

[19] Takakura M, Kuroda T (1998). Morphologic analysis of dentofacial structure in patients with acromegaly. Int J Adult Orthodon Orthognath Surg.

[20] Kreitschmann-Andermahr I, Kohlmann J, Kleist B, Hirschfelder U, Buslei R, Buchfelder M (2018). Oral-dental pathologies in acromegaly. Endocrine.

